# Welcome note

**DOI:** 10.4103/0972-124X.44085

**Published:** 2008

Dear ISP members,

Greetings from Head Office,

It is my pleasure to pen a few words as secretary of ISP for the Journal of Indian Society of Periodontology. Publication of the Journal is an essential part of any scientific professional organization. In the true spirit of our founders and distinguished eminent past office bearers, the new team has taken all the necessary steps to keep our organization in track. ISP has always carved its own image among the dental professional bodies. Journal of our society has a vital role in building up a positive image for an eminent scholar and a dedicated person with enthusiasm and strength Dr. D. Arunachalam has been elected as the editor of JISP. I am sure under his stewardship the journal is going to search its newer heights selecting the image of our society. Our Head Office takes all the measures and lends a helping hand to all the members who want to contribute to the journal. I request co-operation from our members in all our activities including the JISP.

I wish Dr. D. Arunachalam and his team all the success ahead in their venture.

Thanking You,

Yours at Sincere,

M. M. Dayakar
Secretary,
Journal of Indian Society of Periodontology
E-mail: smilesindia@gmail.com

